# Miscellaneous

**Published:** 1884-05

**Authors:** 


### Miscellaneous.

CHICAGO MEDICAL COLLEGE COMMENCEMENT.—The twenty-fifth annual Commencement exercises of the Medical Department of the Northwestern University, better known as the Chicago Medical College, were held in the Grand Opera House, on the afternoon of March 25, 1884. Rev. Joseph Cummins, President of the University, presided on the occasion, and conferred the degrees upon the graduating class, which numbered thirty-eight in regular course, one *ad eundem*, and one honorary. After the opening prayer and music, the Dean of the medical Faculty announced the certificates for the under-graduates, certificates for special work in the hospital and dispensary, and the award of prizes. The prizes for the two best theses were taken by C. S. Bacon, of Wisconsin, and George A. Staples, of Iowa. The alumni prize, for the best average scholarship in all the branches of the three years' study, was taken by A. C. Helm, of Illinois; the Senn prize, for the best anatomical preparation, by T. H. Swayne, of Illinois; and the microscope prize, offered by W. H. Bullock, of Chicago, and J. Grunou, of New York, to the candidate for graduation who should sustain the best examination in the theory and practice of microscopy, open for competition to the graduating classes in all the colleges of Chicago, both literary and medical, by C. S. Bacon, of Wisconsin. The practical working of the long college sessions, the thorough grading of classes, and the annual examination of each class in its grade, instituted by this college, is well illustrated by the following remarks of the Dean of the medical Faculty, in presenting the present class of candidates for graduation to the President of the University:

"*Mr. President:*—The college class presented by the candidates for graduation before you, entered their first year numbering sixty-five.

"The effect of theoixtanima ensin the branches of medicine embraced in the first year course, was such as to convince fourteen of these that they had better seek some shorter and surer road to the principal object of their ambition, a diploma, and consequently the class in its second year numbered only fifty-one.

"The effect of the examinations in the studies of that year added to the first, produced the same convictions on ten additional members, consequently the class in its third and last year numbered forty-one.

"I regret to say that the result of the third year examinations has caused three (3) of these to be missing from the class as they stand before you to-day.

"These facts will justify me in assuring you that the 38 young men whom I present before you to-day as candidates for the degree of Doctor of Medicine, and for a formal admission into the ranks of one of the noblest and most responsible of professions, have been subjected to a patient, systematic, and thorough course of medical training in every department, from the elementary work in the laboratories of anatomy, chemistry and histology, to the direct practical study of disease at the bedside of the sick in the hospitals and dispensaries. More than this, they had been well prepared for professional studies by a good general education. Seventeen of the 38 are graduates of literary colleges and universities of the highest standing in our country. The remaining 21 have given the medical Faculty full evidence of possessing a good academic education. As the representative of the Faculty of the medical department of the university over which you preside, I take pleasure in asking you to confer on each member of the class here presented the degree of Doctor of Medicine, feeling a strong assurance that by their attainments, their industry, and their personal integrity, they will prove an honor to their Alma Mater, and a blessing to the communities in which they may live."—*Journal of the American Medical Association.*

---

### ILLINOIS STATE MEDICAL SOCIETY.

The thirty-fourth annual meeting of the Society will be held in Chicago, Methodist Church Block, cor. Clark and Washington Sts., beginning May 20, 1884, at 10 A. M.

### OFFICERS FOR THE YEAR 1883–4.

*President.*—Edmund Andrews, Chicago.
*First Vice-President.*—D. S. Booth, Sparta.
*Second Vice-President.*—G. W. Nesbitt, Sycamore.
*Treasurer.*—Walter Hay, Chicago.
*Permanent Secretary.*—S. J. Jones, Chicago.
*Assistant Secretary.*—J. F. Todd, Chicago.
*Members of Judicial Council.*—One Year, C. Truesdale, Rock Island; C. Goodbrake, Clinton; A. K. Van Horn, Jerseyville. Two Years, E. Ingals, Chicago; F. B. Haller, Vandalia; Wm. Hill, Bloomington. Three Years, Robert Boal, Peoria; Herbert Judd, Galesburg; L. G. Thompson, Lacon.

### STANDING COMMITTEES.

Practice of Medicine.—J. C. Frye, Peoria; J. W. Hensley, Yates City; N. S. Read, Chandlerville.

Surgery.—Roswell Park, Buffalo; David S. Booth, Sparta; J. D. Whiting, Petersburg.

Obstetrics.—S. K. Crawford, Monmouth; E. A. Ingersoll, Canton; C. DuHadway, Jerseyville.

Gynæcology.—W. S. Caldwell, Freeport; A. C. Corr, Carlinville; A. F. Rooney, Quincy.

Ophthalmology and Otology.—J. P. Johnson, Peoria; Robert Tilley, Chicago; P. H. Garretson, Macomb.

Drugs and Medicines.—B. F. Crummer, Warren; J. H. Robinson, Chicago; T. M. Collimore, Concord.

Necrology.—E. Ingals, Chicago; William Hill, Bloomington; W. West, Belleville.

SPECIAL COMMITTEES.

Oral Surgery.—J. V. Black, Jacksonville.
Tetanus—.C. Truesdale, Rock Island.
Vaccination.—M. F. Bassett, Quincy.
Diseases of Children.—J. P. Matthews, Carlinville.
Physiology.—A. Wetmore, Waterloo.

[Continued from last year.]

Committee on Legislation for the Insane—Walter Hay, Chicago, Chairman; E. P. Cook, Mendota; Wm. Hill, Bloomington.

The Diagnostic Peculiarities of Malignant Growths.—Christian Fenger, Chicago.

Analysis of a Certain Class of Remedies, Concerning which Physicians are not Positive as to their Therapeutic Value.—W. M. Ransom, Roscoe.

Simple Renal Catarrh.—I. N. Danforth, Chicago.

COMMITTEE OF ARRANGEMENTS.

D. W. Graham, Chairman; C. G. Smith; R. C. Hamill; H. A. Johnson; E. Ingals; J. S. Todd, *ex-officio*.

A full and interesting meeting is anticipated, and the Committee of Arrangements are completing plans for reduced railroad fares, a banquet, and other attentions.

### OFFICIAL LIST OF CHANGES OF STATIONS AND DUTIES OF MEDICAL OFFICERS OF THE UNITED STATES MARINE HOSPITAL SERVICE, JANUARY 1, 1884, TO MARCH 31, 1884.

Fessenden, C. S. D., Surgeon, to proceed to Cairo, Illinois, and Memphis, Tennessee, as inspector, March 5, 1884.

Purviance, George, Surgeon, granted leave of absence for thirty days, Feb. 16, 1884.

Smith, Henry, Surgeon, to rejoin his station at Norfolk, Virginia, March 7, 1884.

Irwin, Fairfax, Passed Assistant Surgeon, relieved from duty at Norfolk, Virginia, to assume charge of Cape Charles Quarantine Station, March 7, 1884.

Carmichael, D. A., Assistant Surgeon, to report to Surgeon Purviance for examination for promotion, March 5, 1884.

Armstrong, S. T., Assistant Surgeon, to report to Surgeon Fessenden for examination for promotion, March 5, 1884.

Bennet, P. A., Assistant Surgeon, leave of absence extended ten days, January 18, 1884.

Ames, R. P. M., Assistant Surgeon, detailed for temporary duty on relief boat—Ohio River flood sufferers, February 16 and March 1, 1884.

Devan, S. C., Assistant Surgeon, upon expiration of leave of absence, to proceed to St. Louis, Mo., for temporary duty, February 6, 1884.

Kalloch, P. C., Assistant Surgeon, to proceed to Charleston, S. C., for temporary duty, February 1, 1884.

Bevan, A. D., Assistant Surgeon, granted leave of absence for seven days, March 13, 1884.

Wasdin, Eugene, Assistant Surgeon, granted leave of absence for fifteen days, March 4, 1884.

Battle, K. P., Assistant Surgeon, to proceed to New York, N. Y., for temporary duty, February 4, 1884.

### RESIGNATION.

Cook, H. B., Passed Assistant Surgeon, resignation accepted by the Secretary of the Treasury, to take effect February 5, 1884. January 31, 1884.

### APPOINTMENT.

Battle, Kemp P., M.D., of North Carolina, having passed the examination required by the Regulations, was appointed an Assistant Surgeon by the Secretary of the Treasury, February 2, 1884.

### PROMOTIONS.

Carmichael, D. A., Passed Assistant Surgeon, promoted and appointed Passed Assistant Surgeon, by the Secretary of the Treasury, from March 1, 1884. March 18, 1884.

Armstrong, S. T., Passed Assistant Surgeon, promoted and appointed Passed Assistant Surgeon, by the Secretary of the Treasury, from April 1, 1884. March 28, 1884.

